# The role of health protection teams in reducing health inequities: findings from a qualitative study

**DOI:** 10.1186/s12889-023-15143-7

**Published:** 2023-02-02

**Authors:** Rosalie Allison, David J Roberts, Adam Briggs, Shona Arora, Sarah Anderson

**Affiliations:** 1 Programmed Delivery Unit, Health Security Agency, London, UK; 2 Health Protection Team, Health Security Agency, South East, UK; 3 Health Equity and Clinical Governance, Health Security Agency, London, UK

**Keywords:** Inequalities, Disparities, Behavioural science, Intervention, Role, Public health, Communicable diseases, Interviews, Focus groups, Strategy

## Abstract

**Introduction:**

The UK Health Security Agency’s (UKHSA) Health Protection Teams (HPTs) provide specialist public health advice and operational support to NHS, local authorities and other agencies in England. The development of a three-year UKHSA Health Equity strategy creates a unique opportunity for HPTs to reduce health inequities within their work.

**Aims:**

This study aimed to understand current health equity activities and structures within HPTs, and to propose future HPT-led health equity activities.

**Methods:**

Between November 2021 - March 2022, HPT staff from the nine UKHSA regions were invited to participate in a semi-structured interview or focus group.

**Results:**

Twenty-seven participants covering all nine UKHSA regions took part in a total of 18 interviews and two focus groups. There was enthusiasm to address health inequity, and many reported this as their motivation for working in public health. All HPTs routinely engaged in health equity work including, variously: liaising with other organisations; advocacy in case and outbreak management meetings; developing regional HPT health equity action plans; and targeting under-served populations in day-to-day work. HPT staff discussed the challenge of splitting their time between reacting to health protection incidents (e.g., COVID as the main priority at the time) and pro-active work (e.g., programmes to reduce risk from external hazards for vulnerable populations). Although COVID had raised awareness of health inequities, knowledge of health equity among the professionally diverse workforce appeared variable. Limited evidence about effective interventions, and lack of clarity about future ways of working with other organisations were also shared as barriers to tackling health inequities.

**Conclusion:**

HPTs welcomed the development of UKHSA’s health equity strategy, and through this study identified opportunities where HPTs can influence, support and lead on tackling health inequities. This includes embedding health equity into HPTs’ acute response activities, stakeholder working, and staff management. This study also identified a need for health equity training for HPTs to improve knowledge and skills, utilising evidence-based approaches to health equity. Finally, we have identified areas where HPTs can lead, for example using brief advice interventions and through developing resources, such as standard operating procedures that focus on vulnerable populations. These findings will support a more integrated approach to addressing health equity through health protection work.

**Supplementary Information:**

The online version contains supplementary material available at 10.1186/s12889-023-15143-7.

## Background

The World Health Organisation (WHO) defines health equity as the “absence of unfair, avoidable or remediable differences among groups of people, whether those groups are defined socially, economically, demographically, or geographically or by other dimensions of inequality (e.g. sex, gender, ethnicity, disability, or sexual orientation). Health is a fundamental human right. Health equity is achieved when everyone can attain their full potential for health and well-being.” [1].

Health inequities may be driven by:

- different experiences of the wider determinants of health, such as the environment, income, or housing.
- differences in health behaviours or other risk factors, such as smoking, diet and physical activity levels.
- psychosocial factors, such as social networks and self-esteem.
- unequal healthcare access, experience, or outcomes [2].

The COVID-19 pandemic has exposed and exacerbated inequities in society [3–14]. Often the communities that are least able to cope have suffered the most, both from the disease, as well as through the indirect effects of policies aimed at containing viral spread [15].

Health protection issues, such as low vaccine uptake, infectious diseases (e.g., Tuberculosis (TB) and Hepatitis C) and antimicrobial resistance (AMR), disproportionately affect inclusion health groups (e.g. some migrant groups, people in contact with the criminal justice system, people who misuse drugs or alcohol, those who are homeless) or other at-risk groups who already experience health inequities (e.g. based on ethnicity or sexual orientation) [16–18]. In regards to health protection hazards, vulnerable populations are at greater risk, due to environmental or behavioural risk factors and also have specific prevention needs which may not be met; additionally, these groups may have poorer healthcare access, experience and outcomes, and lack of social support to enable timely diagnosis and treatment, resulting in further transmission and/or more adverse consequences of disease, and widened inequities [16, 19].

Achieving health equity requires identifying and addressing inequities, wherever they exist. Narrowing inequities is complex and often described as a ‘wicked problem’ requiring system-wide solutions and innovative thinking [20].

The 2010 Marmot Report [20] laid out six policy objectives to reduce health inequalities and a framework for delivering and monitoring reductions in health inequalities along the social gradient. However, Marmot’s 2020 ‘10 years on’ report [21] showed that improvements in life expectancy had slowed dramatically and poor health had increased everywhere. This is concerning, as the substantial negative health outcomes and economic consequences within populations are generally avoidable, with targeted evidence-based interventions.

Current strategies and approaches in England include:

- The NHS Long Term Plan (NHS LTP) which sets out an objective on prevention and health inequalities, with a focus on reducing local health inequalities and unwarranted variation [22, 23].
- NHS England and NHS Improvement’s Core20PLUS5 approach to support the reduction of health inequalities at both national and system level. The approach defines a target population cohort for action that includes the most deprived 20% of the national population (the ‘Core20’) and inclusion health groups and protected characteristic groups (PLUS). The ‘5’ refers to focus clinical areas requiring accelerated improvement [24].
- The government’s ‘Levelling Up’ White Paper which has a mission to: narrow the gap in Healthy Life Expectancy (HLE) between local areas where it is highest and lowest, by 2030; and raise HLE by 5 years, by 2035 [25].

The 2021 public health reform dissolved Public Health England (PHE) (an organisation whose aim was to protect and improve the nation’s health and wellbeing, and reduce health inequalities [26]), and created the Office for Health Improvement and Disparities (OHID) and the UK Health Security Agency (UKHSA). OHID’s focus is on improving the nation’s health so that everyone can expect to live more of life in good health, and on levelling up health disparities to break the link between background and prospects for a healthy life [27]; and UKHSA is responsible for protecting every member of every community from the impact of infectious diseases, chemical, biological, radiological and nuclear incidents and other health threats [28].

The UK Health Security Agency’s (UKHSA) Health Protection Teams (HPTs) provide specialist public health advice and operational support to the NHS, local authorities, and other agencies. HPTs also prevent and reduce the effect of diseases, chemical and radiation hazards, and major emergencies, through: local disease surveillance; maintaining alert systems; investigating and managing health protection incidents and outbreaks; and implementing and monitoring national action plans for infectious diseases at local level [29].

The UKHSA’s remit letter states that, to support delivery of the Department for Health and Social Care (DHSC)’s approach to health disparities, the organisation has a role in co-ordinating with partners to ensure all members of the community are, as far as possible, equally protected from health threats. As part of its core activities, UKHSA will develop and implement an internal UKHSA health equity strategy, supported by the newly established health equity division [30]. This reveals a unique opportunity for UKHSA’s groups, divisions, and teams to inform the development of UKHSA’s health equity strategy and priorities, including Health Protection Teams (HPTs).

## Aims

This study aims to collate the views of Health Protection Teams, and explores:

- Current health equity activities and structures within HPTs.
- Desired future health equity activities.

The findings will be used to inform national and regional health protection strategies to tackle inequities.

## Methods

### Recruitment and sampling

Initially, a named Health Equity Lead from all nine regional Health Protection Teams in England, were invited to either a 1–1 (interview) or group (focus group) discussion with the lead researcher (RA), depending on participants’ preference. Subsequently, any colleagues recommended by the Health Equity Leads were also invited to participate.

### Interview schedule

The semi-structured interview schedule (Supplementary 1.), developed using the Theoretical Domains Framework (TDF) ( a behavioural science tool used to identify and describe factors that influence a behaviour) [31], covered:

- Roles, responsibilities, and the structure of the team in relation to tackling health inequities.
- Use of strategies / guidance / tools / continuing professional development, aimed at tackling health inequities.
- Priority population / disease groups.
- Barriers and facilitators to tackling health inequities.

In practice, the terms health disparity, inequality and inequity are often, albeit incorrectly, used interchangeably. At PHE the agreed terminology in this space was ‘health inequalities’, however, to better describe the ambition of the UKHSA, the term ‘health equity’ was adopted. As such, interviewees’ responses often shifted between these terms as these new organisations were formed and settled.

### Interview sessions

Discussions were conducted by RA over Microsoft Teams at a time convenient to the participant(s). Participants were reassured that there was an understanding that the organisation was going through a period of change, and that there was no expectation regarding current health equity activities within their HPT. Participants were also reassured that the transcripts would be anonymised, so they could be as open with their opinion and share as much as they felt comfortable with. The interviewer (RA) was an experienced researcher, with a background in Public Health, but no experience of working in Health Protection Teams. On average, discussions lasted approximately 50 min.

### Analysis

With the participants’ consent, the discussions were recorded and transcribed, verbatim. Transcripts were checked for accuracy by RA and anonymised. RA thematically analysed the transcripts [32] and mapped the main themes to the Theoretical Domains Framework using NVIVO 11 [31]. Half-way through data collection, the existing anonymised transcripts were randomly circulated to a small group (including RA, SA, DJR, as well as three other public health colleagues) to review and discuss: the main themes; suggested implications of the findings; any recommended changes to the interview schedule for the remaining discussions.

All UKHSA participants were invited to a follow-up workshop to review the main findings and discuss future priorities for national strategy and regional HPTs’ business plans.

## Results

Between November 2021 – March 2022, twenty-seven participants from all nine UKHSA regions took part in the study (Table 1.). Participants were mainly health protection consultants (14), and health protection practitioners (4). One focus group was with a health equity working group, including OHID regional health and wellbeing colleagues.

**Table 1:** Breakdown of Participants’ Characteristics (N = 27)

Characteristic	Variable	Number of Participants (N = 27)
UKHSA Region	National	2
	East Midlands	1
	East of England	2
	London	8
	North East	1
	North West	2
	South East	1
	South West	2
	West Midlands	7
	Yorkshire and the Humber	1
Job Title, Organisation	Consultant in Health Protection (CHP) / Public Health, UKHSA	14
	Health Protection Practitioner, UKHSA	4
	Epidemiology Analyst, UKHSA	2
	TB Programme Manager, UKHSA	1
	Colleagues from the Health Equity Directorate, UKHSA	2
	Programme Director, OHID	1
	Consultant in Health and Wellbeing, OHID	1
	Programme Manager, OHID	1
	Public Health Registrar, OHID	1
Age	18–24	0
	25–34	2
	35–44	4
	35–45	1
	45–54	3
	45–55	4
	55–64	3
	65+	0
	Blank	10
Gender	Man	8
	Woman	19
	Non-binary	0
	Prefer to self-describe	0
Data Collection	Interview	18
	Focus group (n = 2)	9

The main themes that emerged from the discussions are outlined below. These were reviewed in a follow-up workshop where 85% of attendees voted that they mostly agreed (41%; 13/32) or fully agreed with the findings (44%; 14/32).

### Structures, roles and responsibilities

Participants felt that the remit of the UKHSA was to achieve health equity by considering and accounting for health inequities in their health protection response work, but it was not felt that it was necessarily the role of HPTs to directly address the wider determinants of health inequities. Participants expressed that they felt their role was about finding the balance of reducing disease transmission compared to the risks of mitigation strategies required to do so. Participants felt that, although they were clear of the end goal, health equity was not always routinely considered in HPTs’ work.

*“Realistically, I think the majority of the time, actually what we’re really talking about is equity. So, if we’re looking at people who are intravenous drug users, or people who are homeless, we’re almost certainly going to have to go that additional mile. We’re going to be doing things with them that we wouldn’t do with other people, so it’s not equal, but it’s equitable to make sure that they’re getting the same experience as other people, and that we’re striving to have the best, the same health outcomes for them all.” (CCDC-I-17122021B)*.

*“I remember one of my colleagues saying to me that our job is just to reduce and stop infectious disease spreading. And I don’t believe my job is to stop infectious disease spreading at all costs, it is to balance the risks and reduce the risk of infectious disease against those other risks. So, in schools, the risk of reducing infectious disease by sending all the kids home is not worth it for the impact it has on inequalities.” (CCDC-I-18,012,022)*.

*“…address the impacts rather than the underlying causes. Our service isn’t able to address the underlying causes of health inequalities… I mean, we can highlight the need for tackling the inequality from a Health Protection perspective, but we’re not the providers of accommodation or income or healthcare, so we can’t directly impact that; we could highlight that it’s an issue that needs to be addressed.” (CCDC-1-17122021 A)*.

There was an appetite and enthusiasm among participants to address health inequity, and many participants reported this as their motivation for working in public health. Generally, participants felt that there was interest from other team members too, but some were concerned about staff time and capacity to focus on health equity.

Although there were many differences, there was general agreement that HPTs have a role in:

- liaising with other organisations / agencies to tackle inequities.
- advocating for health equity in outbreak / incident management meetings.
- developing regional HPT health equity action plans.
- managing day-to-day incidents, with extra effort put into reaching under-served populations.
- pro-actively working with identified inclusion groups to understand their lived experiences and co-develop interventions to address inequities.

HPT staff discussed the challenge of splitting their time between managing health protection incidents (e.g., COVID as the main priority at the time) and pro-active work (e.g., programmes aimed at reducing risk from external hazards for vulnerable populations, including migrant health, Tuberculosis (TB), Sexually Transmitted Infections (STIs), migrants and asylum seekers, early years and school-aged children, people in contact with the criminal justice system). Staff members also had roles as geographical patch leads (leading for UKHSA on activity in a local authority area), managing local health protection incidents and strategic work with local stakeholders, such as local authority public health.

*“The core team, which is made up a mixture of consultants and practitioners…where we’re all providing an acute response, and we do that by a single acute desk. Practitioners are likely to spend the majority of their time ….on the acute desk, and then they will have a range of programmes that they feed into, and one of those programs is the inequalities programme… Sounds marvellous until you bring in the fact that our practitioners are constantly told to deprioritise programmes and prioritise the desk.” (CCDC-1-18012022)*.

In relation to health equity activities, there was a full spectrum, in terms of progress, set-up and activities officially labelled as health equity projects. For example, where some HPTs already had a health equity team within their regional HPT, there was a desire from other HPTs to set this up, with a named champion within each patch. At the time, one HPT had a health inequities group which also included their OHID regional health and wellbeing team, which was considered advanced progress.

“Within **[region] there are three Health Protection teams. There are periods of time and elements of work which we do across **[region]. So, for example all our SOPs around how we manage a particular infection or how we manage a particular situation are developed within a Pan **[region] group with representation from each of the three HPTs. However, each of the three HPT works a little bit differently and takes on different projects, and cover different populations, so there will be slightly different issues within each of those areas. And so, from the point of view of health inequalities, we hadn’t, across **[region], until late last year, really had a formalised health inequalities group and then the convening of that first national meeting, kind of pushed us to do that. So, all three of the HPTs had done various bits of work or had more, or less, formalised programmes of work with regards to health inequalities.” (CCDC-FG-24,012,022).

“My honest opinion is that people are doing it and not realizing they’re doing work towards it… As an example, avian flu, we have had quite a lot of it in **[location], and there is a specific sort of population of people who end up doing a lot of the sort of nasty work with avian flu, picking up dead birds, and that sort of thing, and that population tends to have sort of worse health outcomes, they’re in poorer health, they’re less likely to take Tamiflu, they are managed by a different organization, no proper occupational health, disengaging… There’s a fair bit on health inequalities here that needs addressing, even if it’s not labelled as such, if that makes sense. And so, I think that that goes for health practitioners, business support as well, who are on the phones. Now, what I think, potentially we could do, is expand that and recognize what we’re doing, and in recognizing it and labelling it, you enable people to do it more.” (CCDC-I-19,012,022).

There were also differences in team staffing as some HPTs reported access to data and surveillance expertise that could provide intelligence, to inform action and decision making, which was considered beneficial.

Participants acknowledged that health inequities were a clear thread in public health curricula, but recognised that, post-COVID, HPTs and the wider UKHSA were more professionally diverse than before the UKHSA formed, resulting in mixed awareness and knowledge of health inequities.

(See Supplementary 2. for additional quotes and examples related to ‘Structure, Roles and Responsibilities’).

### Priority population / disease groups

There was a general agreement to move to a more holistic approach to support populations most in need, rather than siloed disease / hazard-specific working. Table 2. shows the groups that participants reported requiring the most focus to address health inequities, and the diseases / health protection issues they most associated with health inequities. (See Table 3. and Supplementary 3. for case studies of health protection health equity activities).

**Table 2:** HPTs’ views on population and disease / health protection issues to prioritise health equity action

Priority population groups	Priority diseases / health protection issues
Migrant health Minority ethnic groups Homeless Those with no recourse to public funds Substance misuse / injecting drug users Gypsy, Roma and Traveller communities Sex workers Men who have sex with men People in contact with the justice system Orthodox Jewish groups Care home residents Those in shared accommodation Farm workers Schools / children and young people People with learning disabilities	TB Vaccine preventable diseases COVID-19 Viral hepatitis (A, B, C) STIs and BBVs GIs i-GAS AMR Respiratory infections Avian flu Scabies Air Quality

**Table 3:** Quotes and examples related to ‘Priority Population / Disease Groups’, including examples of health equity activities

Quotes / Examples - Priority Population / Disease Groups	
*“We could do some prevention work that stops anybody getting ill and I think that if we then take a focus on particular populations at risk, which would be relatively straightforward to do, we could do a lot more prevention and joining up. We treat hepatitis B, and we treat invasive bacterial infections, and we’ll treat them all differently. It’s the same population that keeps popping up for all those infections. We could stop talking about infections and start talking about people and populations, and do something there.” (CCDC-I-18,022,022)* *“So, one of our priorities, and these were all linked to the national TB strategy. So, the first one was around underserved population groups. So, we had a workstream looking at prison pathways. Improving detection of active disease and then looking at education and awareness and establishing a prison TB nurse network. We set up a network and working group and did some education and training sessions.” (PGM-I-09032022)* *“Hep C elimination programme is funded by NHS England specialised commissioning, and they’re funding really focused activities to look at hep C elimination. Well, it’s the same risk group for TB, for Hep B, for HIV. So, there’s a real opportunity in there to look at, if instead of screening for one disease, so people not pathogens, you know they’re all saying that aren’t they at the moment. So, if we’re screening somebody for Hep C, why don’t we look at being able to screen for more than that?… You know, to look at it more holistically.” (PGM-I-09032022)* *“We’re doing some work around Hep C elimination. So, a really tiny amendment to the SOP, in terms of, referring people for Hep C screening. It wasn’t there before; great opportunity. We’re also doing some work around when people are in hospital and it’s evident that they are an injecting drug user. Just a quick note to the presiding clinician “can you check that they’ve gotten Naloxone please?” I mean, how long does that take? It’s 15 s. It’s not obviously Health Protection work, but actually it’s, if you want to talk about sort of making every contact count, that’s a tiny tiny bit of our time for what is much more likely to save a life than the prophylaxis of meningo contacts, for example.” (CCDC-I-18,012,022)* *“Many people live in houses in multiple occupation. And I think that’s probably an inequality we haven’t touched on, like the whole kind of, what is a household contact when you live with ten people you’ve never met, you don’t even know their first name, but everyone’s like leaving their razor in the bathroom? That’s another whole, there’s an opportunity we could make sure our guidance looks at houses of multiple occupation and things like that.” (CCDC-I-02032022)* *“That was a big measles outbreak and so there was a lot of targeted work done with one of the consultants who had really close links to that community. She worked very closely with one of the rabbis, who led a lot of work in that community to increase vaccination rates.” (CCDC-FG-24,012,022)*	

### Monitoring and measuring impact

Generally, participants found responding to questions about monitoring progress towards health equity challenging. Their reasoning for this was that health equity objectives had not previously been explicitly set within health protection, and therefore, it had probably never been measured before in their work. They recognised that demonstrating progress on defined outcomes (e.g., vaccination uptake) would take time and that data was crucial to monitor it. Participants suggested that looking at the existing metrics on published data sources e.g. OHID’s Fingertips [33], would be a useful starting point, and then considering what data is relevant for particular populations, and what HPTs could impact. The example of vaccination uptake was a common suggestion.

*“The best thing to do, for the start, would be to review what metrics we already have nationally, and so using the fingertips, etc. and saying, which of these indicators do we have an impact on? So, you know, we don’t have an impact, for example, on number of children in poverty. That’s not something really that the Health Protection team can add any weight on. But we do have an impact on immunization rates… And let’s review them more regularly for our boroughs. So, you know, that tool is really good, actually, isn’t it, for being able to compare geographies? And we could just, as a matter of routine, once every six months, or once a year, look at where our boroughs sit, compared to other things, and come up with a sort of action plan about where we focus our attentions according to those metrics. I think that would be the start. Don’t reinvent the wheel. Those metrics have been thought out very carefully, but there are probably new metrics also and some of them are probably quite easy to think about, and sort of more audit type metrics. I mean, even reviewing the number of situations that we’ve managed over a time period which affect a specific group.” (CCDC-FG-24,012,022)*.

Other suggestions for ways to measure and monitor progress in the HPT health equity arena was through auditing HPTs’ case and incident management against agreed standards to understand whether cases were managed according to guidance and whether standard operating procedures (SOPs) could be improved to better consider health inequities. Participants suggested that the relevant fields to facilitate this and other quality improvement activities and research should be built into the new HPT electronic case and incident management system currently being developed (CIMS).

*“In terms of measuring impact, proving that impact, I think there does need to be quantitative measure… And actually, it’s made a real difference… as a result of doing what we’re doing, we’ve got this many people in to stop smoking services.… this is the percentage change in intravenous drug use and homeless people with TB adhering to treatment. I think the difficulty is that the baseline for that doesn’t exist at the moment, so it’s around kind of getting some of that baseline. And maybe it is around kind of recording that, but it has to be done in a simple way… maybe looking at things like CIMS [UKHSA’s Case and Incident Management System] for example. I’m thinking about like really quick checkboxes for example, that people could do within the programme that says, you know, have you done XY&Z and then we could kind of have a look at the outcomes of that. Working perhaps with local authorities around gathering, so if we are referring into services, for example kind of trying to get some of that information back. So how many times have Health Protection referred into our services and what has that resulted in? I guess, kind of linking up some of those data streams.” (CCDC-I-17122021B)*.

Participants suggested that they would consider that their actions had made a difference to health inequity if there were changes in national guidance, such as national guidance on diagnosing measles, rash in pregnancy, chicken pox, hepatitis B etc., to include how rashes or jaundice will appear on various skin types or amending the population groups recommended Hep B vaccinations (See Supplementary 2. for additional quotes and examples related to ‘Monitoring and Measuring Impact’.)

### Challenges and Future Direction

Resources, staffing levels, time constraints and organisational barriers, were mentioned as factors that impact the ability of staff to tackle health inequity. Participants reflected on these issues and suggested solutions to overcome them.

See Tables 4 and 5 for challenges and recommendations for health equity activities within Health Protection Teams’ remit.

**Table 4:** Challenges and recommendations / future direction for health equity activities within Health Protection Teams’ remit

Challenges	Recommendations and future direction
Following transition, HPTs are not yet clear on UKHSA and HPTs’ health equity remit; and future ways of working between key organisations	National strategy, with a focus on local implementation, covering: remit, expectations, workforce, impact indicators Cross-governmental / sector commitment, with senior leadership buy-in, could strengthen implementation
Thought on priority groups were generally subjective, based on what had been coming across the acute response desk, rather than any systematic analysis	Take a population focus on the most marginalised / underserved groups
HPTs already work to address health inequities, but it is not recognised or considered in guidelines, ways of working, job role etc.	Embed health equity in all that HPTs do e.g., SOPs, guidance, audit, clinical reviews, outbreak report, risk assessments, job roles, commissioning contracts
Lack of evidence about effective interventions	Embed evaluation into project plans Share good practice through appropriate networks
Data systems, data collection and access to data on key marginalised and underserved populations is a challenge.	Identify what population data is needed and advocate for these to be part of routine data collection. This could also include prompts for HPTs, and automated flagging processes with relevant teams, if certain criteria are ticked
HPT staff split their time between reacting to health protection incidents (e.g., COVID as the main priority at the time) and pro-active work (e.g., programmes aimed at reducing risk from external hazards for vulnerable populations, including migrant health, TB, STIs, early years and schools, health and justice). This is also linked with having the time and capacity to focus.	Health Equity Champions within each region with protected time
Mixed awareness / knowledge of health equity in the professionally diverse UKHSA workforce.	Skills audit of current staff Health equity training for HPTs to improve knowledge and provide specific skills relating to evidence-based approaches to health equity Clear and frequent comms, for the public and healthcare professionals

**Table 5:** Quotes and examples related to ‘Challenges and Future Direction’

Quotes / Examples - Challenges and Future Direction	
*“PHE was dismantled to UKHSA, where the focus is more on Communicable Disease Control, the pandemic and responding to chemicals and radiation. Whereas, the wider team that focused on health inequalities moved into OHID. What we need to be clear in UKHSA, what is our remit and how we respond to it and how do we work in partnership with both OHID and DHSC? And at a local level, how do we work in partnership with NHS and the local government where there are already existing good partnership working?” (CCDC-I-06012022)* *“Let’s say we’ve got an i-GAS outbreak in injecting drug users. We’ve got the people that we know well, up in Colindale, who we can talk to about i-GAS, who’ve got lots of experience with injecting drug users; but actually, there’s a whole substance misuse team who’s now gone outside our organization, who we really should engage, because they’ve probably got lots of links into third sector and on the ground organisations who can help. So, I do worry a bit about that being an issue with the transition and how we make sure that we don’t lose those connections.” (CCDC-FG-24,012,022)* *“The way we are the UK Health Security Agency. People who feel they may be victimized may be less likely to engage with an organization which has security in its title. If they feel that they’re more likely to suffer prejudice. I think that’s a barrier that the organization needs to consciously address.” (CCDC-I-17,122,021 A)* *“So, for people with learning disabilities, in my dreams, before I retire, there would be a Green Book recommendation that kids that bite recurrently get vaccinated against Hep B. And they probably ought to be agreeing that recommendation about young people in the care system being vaccinated.” (CCDC-I-02032022)* *“Probably, the bigger challenge is the evidence on measures to address health inequalities. One of the things with health inequalities is the, because these are often societal impacts, it’s not so straightforward to come up with initiatives to address those that can be measured in our classic medical evidence-based manner. You can’t really do a randomized controlled trial, or the ethics would be incredibly difficult to have a randomised control trial.” (CCDC-I-17,122,021 A)* *“I am currently the only person who is working on health inequalities… what I’d like to do is to create a network of people who are focused on this. I mean, for me, health inequality is a cross-cutting theme, and it should be included in everyone’s job role. I think we are so far away from that in the [Region] that my initial thing is to have a group of people working on it. So, it’s not just me. I genuinely don’t believe that one person can do all this. I need people, particularly within each of the patches, to be able to kind of be disseminating this work, to be able to feed things back in etc. So that is the next step for me.” (CCDC-I-17122021B)* *“* ***As you said at the beginning, you’ve worked in this area for a long time. What’s the knowledge and experience of other people on the team?*** *I think that’s a good question. I think it’s going to be very variable, I would say, depending on people’s backgrounds. We’ve all come from such a wide variety of backgrounds, and I think people who are new to health protection will often have had a lot of, kind of, on the ground experience of it, for example in their different roles before, in nursing or paramedic or environmental health. But wouldn’t necessarily be, I would think, familiar with the sort of evidence base, the sort of frameworks and the tools. I think it’s very variable.* ***Are you doing any kind of local education for them, or do you think there’s a need for that to be centralized some way?*** I think it would be really good to do that. I think having something locally would be good, or **[Region] wide so that we can think about applying that into our work. But if there was some sort of national, I don’t know, national resources that we could use rather than us having to put something together ourselves. I think that would be really good actually. ***Sure. What kind of things would be helpful to cover in that, do you think?*** *I think some of the basics of the sort of evidence base, the way that you can think about inequalities, the various sort of frameworks and models. But then, somethings that are really practical examples of where, where we might come across this, because, I think, sometimes we don’t always think about it.” (CCDC-I-10,032,022)* *“I’m really keen to both draw on the benefits of having fished from a wider pool, but also think about what the needs of that team have been. I think there’s probably a lower concentration of people with Master of Public Health than there has ever been before, that kind of thing. So actually, what is their understanding of inequalities? How many times has someone spoken about wider determinant with them and then balance that with thinking about what brief intervention looks like for an acute desk, because it doesn’t look like talking to somebody about their weight, it just doesn’t… But we constantly have to talk to people about their vaccination status. We’re calling it a skills audit, looking at what our team have, what they bring, what we can utilize, and then where we need to fill the gaps.” (CCDC-I-18,012,022)*	

## Discussion

### Summary

UKHSA Health protection teams see that they have a role in addressing health equity going forward, both within their reactive management of health protection incidents and outbreaks, as well as with their pro-active project and stakeholder working. At the time of data collection, challenges to this included: time and capacity to put the processes in place to embed health equity; lack of evidence about effective interventions for health protection; challenging systems, data collection and access to data; mixed awareness / knowledge of health equity in the professionally diverse, post-COVID HPT workforce; uncertainty in the UKHSA and HPTs’ health equity remit, and future ways of working between key organisations.

### Comparison to other literature and recommendations

It is known that public health agencies have a fundamental role in understanding the health needs of deprived communities and inclusion health groups, identifying interventions to improve their health and providing leadership at local and national levels [19]. However, to our knowledge, this is the first study explicitly exploring the involvement of Health Protection Teams in England to deliver the health equity agenda.

HPTs identified a role in pro-actively working with inclusion health groups to understand their lived experiences and co-develop interventions to address inequities. This community-centred approach is consistent with the published place-based approach to tackling health inequalities [34]. However, HPTs felt that the evidence behind effective interventions were lacking, which was corroborated by the 2018 systematic review and meta-analysis of morbidity and mortality in homeless individuals, prisoners, sex workers, and individuals with substance use disorders [35]. The review recommended that consistent data collection will allow public health agencies to develop, implement, and evaluate structural interventions that improve the health of inclusion health groups [19]. Saini et al. support the value of involving patients and public in research and evaluation [36].

The King’s Fund state that “now is the time to embed work to address health inequalities” [37]; these views were also shared by HPTs who expressed that, given the importance of tackling health inequities to protecting health, now is the time for collective action to address health equity. Given the recent changes to the public health system in England, there is a real opportunity to inform development of the health protection teams’ role. The continued importance of addressing health inequity is also evidenced by explicitly considering health inequalities in the UK COVID-19 Inquiry, which aims to examine the UK’s response to and impact of the COVID-19 pandemic, and learn lessons for the future [38].

There is agreement in the literature that population health concepts should sit at the core of health curricula [39], with a focus on inclusion health groups [40–42]. However, due to the multi-disciplinary background of the public health workforce, development of a health protection-specific health equity training is recommended. Any education or training developed could be based on existing resources and evidence-based approaches and tools, such as All Our Health [43], Health Equity Assessment Tool (HEAT) [44], or Making Every Contact Count (MECC) [45].

### Strengths and limitations

The main strengths of this study are the robust methodology, underpinned by behavioural science; and the timeliness of the discussions, to capture the views of HPTs shortly after transition from PHE to UKHSA, which can be used to inform development of the UKHSA health equity strategy. Further studies could go further to address and unpack some of the themes and the relative impact HPTs might make addressing health inequalities through different mechanisms e.g. on wider determinants.Furthermore, although there were more participants from London and the West Midlands, these teams covered some of the largest populations, and had many smaller areas within their regions; therefore, it was appropriate to collect the views from colleagues across the region, as recommended by the regional health equity lead. Moreover, participants from health protection teams from all nine UKHSA regional teams in England participated in the study, implying that we can be confident that the findings provide a true picture of current approaches to health equity across HPTs. However, the findings are limited to reflecting the English health protection system, and therefore may not be generalisable to other countries. It could also be argued that there were bias in findings as the primary participants were the nominated health equity lead(s) for their health protection team, suggesting that they were already engaged, and could therefore bias the results. However, all health equity lead(s) were given the opportunity to suggest as many other colleagues as they thought could input into the discussion, and every suggestion was invited to participate in the study.

## Recommendations

Acknowledging that resources, staffing levels, time constraints and organisational barriers, following re-organisation of Public Health agencies, all impact the ability of staff to tackle health inequity is important; to overcome them requires leadership, a focus on the most impactful interventions, and efficient cross-organisational working.

Although many challenges, there was an appetite and enthusiasm to address health inequity within health protection, and UKHSA’s HPTs have a role in this. Recommendations include:

- development of a Health Equity Strategy which sets out the remit and expectations of HPTs and the wider UKHSA.
- take a more holistic health protection approach to support populations most in need, rather than siloed disease / hazard-specific working.
- embed health equity into business-as-usual e.g., including / considering in SOPs, guidance, audit, clinical reviews, outbreak report, risk assessments, job roles, commissioning contracts. Approaches to embedding in health equity in national guidance, could include ensuring that all guidance and supporting resources are inclusive in their language and descriptions and promote equitable public health action across all population e.g., national guidance on diagnosing measles, rash in pregnancy, chicken pox, hepatitis B etc., to include how rashes or jaundice will appear on various skin types.
- develop the workforce through skills audit and health equity training to improve knowledge and provide specific skills relating to evidence-based approaches to health equity.

These findings will support a more integrated approach to addressing health equity through health protection work.

## Electronic supplementary material

Below is the link to the electronic supplementary material.

Supplementary Material 1

Supplementary Material 2

Supplementary Material 3

## Data Availability

The data generated and analysed during the current study is available from the corresponding author on reasonable request.

## List of abbreviations

AMR: Antimicrobial Resistance
BBVs: Bloodborne Viruses
CIMS: Contact and Incident Management System
GIs: Gastrointestinal infections
HLE: Health Life Expectancy
HPT: Health Protection Team
i-GAS: invasive Group A streptococcal
NHS LTP: NHS Long-term Plan
OHID: Office for Health Improvement and Disparities
PHE: Public Health England
SOP: Standard Operating Procedure
STIs: Sexually Transmitted Infections
TB: Tuberculosis
UKHSA: UK Health Security Agency
WHO: World Health Organisation
