# Supplementary material for: The role of health protection teams in reducing health inequities: findings from a qualitative study
Source: BMC Public Health. 2023 Feb 2;23:231. doi: 10.1186/s12889-023-15143-7 (PMC9893953; doi:10.1186/s12889-023-15143-7)
Supplement: Supplementary file 1 — Supplementary Material 1 [file 12889_2023_15143_MOESM1_ESM.docx]

# Health inequalities study interview schedule

# (TDF constructs in purple)

### Introduction (please read to the interviewee before the interview takes place)

My name is Rosie Allison and I am interviewing you on behalf of the UKHSA’s Programmed Delivery Unit as part of a study to explore activities in HPTs across England to help address health inequalities. The interviews aim to provide a more comprehensive overview of health inequalities activities and structures within which HPTs are currently operating, and the issues they are currently facing. This will inform how UKHSA’s health equity group can help you to tackle health inequalities.

I would like to ask you about four topics:

- Roles, responsibilities and the structure of your team in relation to tackling health inequalities
- Use of strategies/guidance/tools / CPD aimed at tackling health inequalities
- Priority population / disease groups
- Barriers and facilitators to tackling health inequalities

If you don’t mind, the interview will be audio recorded and I will take a few notes. The notes and recording will be anonymised before we type it up, meaning we will not use your name or any other information that can be used to identify you. We will send you a transcript if you wish, in order to comment on the interview at a later stage, and you are free to opt out at any stage.

[start transcription (and hide) and recording]

Can I check that you have signed the consent form?

## RECORD:

Verbal consent recorded

Written consent received

HPT identifier

Assigned number

Date of interview

Name of interviewer

- Interview

OR

- Focus Group

## Background knowledge

1. Can you tell me what you understand by the term health inequality? (Knowledge)
2. Can you tell me what you understand by the term health equity? (Knowledge)

## Roles and responsibilities

1. In your individual role, to what extent is the issue of health inequalities a priority for you? (Emotion) May you briefly describe your roles, responsibilities and priorities in relation to addressing health inequalities (Social/professional role and identity)
2. Could you tell me a little bit about the structure and roles within your HPT, in relation to addressing health inequalities. Prompt: job roles/titles/named responsible person and or working group etc, links into decision-making/senior leadership team and governance, collaboration with other HPTs (Environmental context and resources)
3. Could you tell me a little bit about people’s attitudes towards health inequalities within your HPT. Prompt: perceived importance (to the practitioners, consultants etc) (Social influences)
4. To what extent do you think you can make a difference to health equality in your HPT role? Probe: what can you (indv and organisation) do to make a difference? (Optimism/ Social/professional role and identity)
5. To what extent do other HPT staff feel that they are making a difference in tacking health inequalities? Probe: what kind of support do you think they need to be able to make a difference? (Beliefs about capability)
6. How confident are you that your HPT is going to make a positive difference to health inequalities this year? (Belief about consequences)
7. How do you think that action by HPTs on health inequalities might be measured? How will UKHSA / HPTs know they are making a difference and reducing HIs? (Behavioural regulation)

## Health Inequalities strategies / guidance / tools / CPD

1. How does your HPT aim to tackle health inequalities? Probe: Is there a HPT strategy or action/work plan? What other local strategies does your HPT have in place? (Intentions)
2. What are the priority population groups / diseases in your region? Why these groups? Probe: based on data / an audit? (Behavioural regulation)
3. Can you tell me which health inequalities initiatives, guidance and tools you (indv) are familiar with? Probe: MECC/brief interventions, HEAT tool, All Our Health, ‘place-based approaches for reducing health inequalities’ guidance, disease-specific health protection guidance. Any locally developed? (Knowledge)
4. What is your opinion on the initiatives you (indv) are familiar with? Probe for each: Have you used them? How have they been locally implemented/adapted? What do you think of them? How useful are they? What works and what does not? (Environmental context and resources)
5. Can you tell me about any educational resources you (indv) know of for your HPT staff around tackling health inequalities? Probe: (Skills) All Our Health, e-learning for health, equality and diversity mandatory training module, Faculty of Inclusion and homeless health, diversity and inclusion type resources and training eg. PHE 2021/22 diversity and inclusion business plan

## Feedback and communication within and between HPTs and other teams / networks

1. Could you tell me a little about any other teams/networks that you may interact with in health inequalities planning? Probe: What currently exists? Who do they involve? Who do they interact with? Lead for different population groups? At what level I.e. regional vs county-level; specifically with OHID, NHS, Local authorities. How has this changed since the transition to UKHSA? (Environmental context and resources)
2. How are you (as a regional HPT) engaging with other teams / networks to make a difference? Prompt: Incentives, audits, feedback, any other? (Reinforcements)
3. To what extent are other teams / networks engaged in addressing health inequalities? What are the difference from PHE to UKHSA? Prompt: people / focus / Are the guidance/ resources / CPD used? (Goals)
4. What motivates other teams / networks to decide to make a difference? Probe: financial incentives, the importance of tackling health inequalities, personal experience, feedback, health inequalities rates (Memory, attention and decision processes)

## Barriers and facilitators for addressing health inequalities

1. What are the barriers to tackling health inequalities that you have encountered? Probe: financial, staff, time, stress, national support (Environmental context and resources)
2. What could facilitate better health inequalities strategies or implementation within your region? Probe: what support do you think your HPT / other teams or networks? Education / training, more staff, resources (Environmental context and resources)
3. Suggestions of other appropriate contacts to interview
