# Supplementary material for: The role of health protection teams in reducing health inequities: findings from a qualitative study
Source: BMC Public Health. 2023 Feb 2;23:231. doi: 10.1186/s12889-023-15143-7 (PMC9893953; doi:10.1186/s12889-023-15143-7)
Supplement: Supplementary file 2 — Supplementary Material 2 [file 12889_2023_15143_MOESM2_ESM.docx]

Supplementary 2. Additional quotes to support the main themes

| Theme | Quote / Example |
| --- | --- |
| Structure, Roles and Responsibilities | “looking at data as best we can in relation to the groups where [vaccine] uptake is lowest. And there’s lots of evidence around that, as well, from nationally and various studies. So, it would be, I suppose, with advocating for that, highlighting that evidence and saying this is where we should focus our activities. Obviously, a lot of what's done, in relation to that, actually is not necessarily us that's doing it. It would be local authorities, the NHS and so on. But I suppose it's that advocacy sort of role and, you know, highlighting things that could be done.” (CCDC-I-10032022)  “So, if you're attached to a geographical, a local authority used to, every year, produce a joint needs assessment for their borough that informs their commissioning plans. And we can feed into that… Into their own local action plan to address the inequalities.” (HPP-I-02022022)  “We all have key patch areas, so we've all got a specific areas within our region that we “look after”, in inverted commas, and we've also got sort of key topic areas and those topic areas include migrant health, health and justice and then other things that are more disease focused, so AMR, TB, STI, hepatitis, whatever and everyone has a topic.” (CCDC-19012022)  “when I was at first a consultant, I was often disappointed at practitioners and even, in some cases, some of the older traditional consultants who had a very narrow view that their remit was purely Health Protection, and so wider health inequalities, wider public health wasn't their concern. I am firmly of the opinion that, as a public health consultant, as public health specialists, which a consultant of Health Protection or a CCDC [consultant in communicable disease control] is, our remit is public health per se. Our current roles focus on Health Protection, but it's our professional role and duty to be mindful of wider Health Protection. There has been a shift in that over recent years. For example, with safeguarding. It's now firmly embedded that safeguarding is an issue that we expect staff to consider. When I first joined the team, there were staff, including consultants, who didn't feel we had any role in safeguarding and I think wider public health and health inequalities is an area where it can be better embedded, recognizing the limitations that our service isn't as directly involved in tackling health inequalities as for example, a local authority public health team. But they are partners of ours and we can help inform their work.” (CCDC-I-17122021A)  “Whenever I speak about health inequality with other people in the team, there is a definite passion for it. People are interested. they think it's important, that's why they came into public health, or part of the reason, it's part of the reason they stay in public health. And so, people are interested in it and want to do something about it, but I think it's too often and too easily forgotten.” (CCDC-I-30112021) |
| Monitoring and Measuring Impact | “For example, if you're looking at TB again, the important thing to look at is travel and migrant population and homelessness and drugs and alcohol abuse. These are the areas where TB continues to flourish, so identifying what that data is relevant for a particular condition or for a particular area and how we access the data and how we make sure that the data is analyzed to see improvement overtime, is a key area UKHSA needs to look into.” (CCDC-I-06012022)  “Yeah, that's a good question. It's very difficult because the actions are unlikely to directly impact on health inequalities in a big enough way, to measure in a short time period. So, you know, it sounds like measuring how many infections, or you know blood borne virus infections we have, you could measure that over years, but it's not going to happen quickly. And I think there's things in terms of how we, the process type measures things we do, and if we do them well. For example, if somebody has a language barrier, do we use a translation service, and do we do that appropriately and, if someone is a person who injects drugs, are they given the right guidance and the right public health advice, reliably. So, we can, at least you know, those are just examples, there's other ways, but we could at least know we're doing the right things well.” (CCDC-I-30112021) |
| Challenges and Future Direction | “I think it's that lack of health inequalities being quite an explicit function within Health Protection. You know, I think we don't think about it enough… I don't think that's always been the way we've culturally done it. I think now, it's an opportunity to reframe it, isn't it, to think differently. I think that's a really good opportunity to actually embed health inequalities and health equity as an important tool, cornerstone.” (CCDC-I-16022022)  “It’s not only our SOPs to be changed, our UKHSA guidance team need to make changes to their documents to include equality and diversity. For example, the national guidelines on Measles, Rash in pregnancy, Chicken pox, hepatitis B, etc need to include how rashes or jaundice will appear on various skin types. The SOPs the Health Protection Team use are summaries of national guidelines. So, the change need to happen from on top as well. Therefore, we need organisational change.” (HPP-I-02022022)  “about LGBT… particularly the national SOPs seem to assume everyone lives in a heterosexual nuclear family, which is so not the case.” (CCDC-I-02032022)  “I think there is something about reflecting in our risk assessments, what are the inequalities and then adjusting our risk assessments. On balance, it's better this school stays open. There is a small risk of D&V, but actually a very high risk of what happens when children don't go to school. I think we should build the inequalities and safeguarding issues much more into our risk assessments.” (CCDC-I-18022022)  “Some of the migrant populations who we serve, who come from countries where government agencies are less trusted and that is a barrier to people's access to healthcare and engagement with organisations such as ourselves, as well, because they may just simply have a low level of trust of government agencies, whatever the government is, because of their previous experience. So that's a challenge to tackle.” (CCDC-I-17122021A)  “You're often dealing with populations who are not as engaged with the health service, who are may be suspicious, understandably of research or of security agencies. The name UK health security agency; I don't know how we can manage health inequalities or how we could even get people to talk to us when we phone up and say that we're from a Security Agency. Most of them are just going to hang up… talking to headteacher about sending out a letter and said, “would you like us to send you a template letter?” I can't even remember what the infection was, and the headteacher said, “I'm not sending anything out with UK health security agency on it. It will scare everyone off. Thank you for the wording, but we will put it on our headed paper”.” (CCDC-FG-24012022)  “The other day we had a situation with someone who was called based on a result that we'd received, and the health protection practitioner called them and identified them by their name that had been passed to us by the NHS. But, actually, it turned out that they were currently in the process of transition and they no longer wanted to be identified by that name, and they were kind of non-binary at this point and had a different name than on the HP zone system. We don't really have anywhere to mark that and I was really concerned with the fact that, if we had to contact that person again we might be in a position where we were going to call them by the same name again and we were going to refer to them by the gender that they were assigned at birth. I don't want to be in this position. So, through that, I was kind of advocating; we're now moving to a new data system and I have therefore spoken to that data system lead and said, listen, this is a situation that we've had, I think it's really important the new system allows us to be able to have preferred name, preferred pronouns, in there for these kind of situations.” (CCDC-I-17122021B)  “Other barriers: an explicit funding envelope. I don't have a budget. Should I have a budget? Can I spend money on training or systems? There's lots of different silos. You know, there's lots of pieces of work going on across the organization, and having a way of knowing I or someone else is feeding inequalities into that.” (CCDC-I-18012022)  “We have access to a lot of information if we want to collect it. We are really restricted at the moment by systems and by data collection. And I think like with anything particularly public health, you need solid data to build the foundation of anything that you are taking forward... I do feel really restricted by how we collect data and how we access data and how we sometimes share data across the organization.” (Epi-I-02032022)  “Tuberculosis is a better example because there is a longer term treatment required for it. How can we support the people who are homeless or who don't have a car to drive to their clinic appointment? How do they get there and how they continue with the treatment for six months? So it is about access, it is about having a vehicle or can we as UKHSA organize transport? If there is no transport to take them to the hospital appointment, can we provide transport? You have to work with the local government, will they be able to get the transport to get them to the hospital appointment. So, these are all probably practical examples of whose remit it is, and where do we have the money or the resources to make it happen? And another example is the refugees and asylum seekers. You know, if there is an issue with their health, how we can get the right support and you always have to rely on, either the local government, or the NHS, because they are the Commissioners of the service and they hold the budget, because we’re not a commissioner of any service, we don't hold any money or budget for anything. So, you know, we are more seen as advisory in that capacity, so I don't know how much of a difference we can make in this situation.” (CCDC-I-06012022)  “The phrasing as well. The terminology, as you said right at the beginning, you know, are we talking about inequalities? Are we talking about equities? Are we talking about disparities? Potentially, they all mean the same thing, but I'm sure that there is some political decisions around why these things are called such things.” (CCDC-I-28022022)  “There is something about going to local communities and different groups and asking them, You know, again, I think we're very good at saying, oh, this is your problem, let's help you sort it or let us sort it out for you. I don't know if it still exists; there used to be an African Health Network in **[HPT] that was really active and they were amazing… They would hold conferences, multi-agency conferences every year or so. They were really very good, and very active in, sort of, getting key messages out and also highlighting particular issues of concern for different communities within the region…For me, it would be understanding who's out there now.” (CCDC-I-16022022)  “Our organisation and teams need to be more diverse and inclusive to reflect the people/community we serve. This includes in our senior management and executive teams.” (HPP-I-02022022)  “It's a very short term, one year strategy, sort of recognizing that in our HPT, lots of people would not necessarily be the health public health backgrounds as they would have done a couple of years back, because, you know, COVID. So, we were seeing it's a workforce development issue. People don't necessarily understand the language and so on. So we put in place a range of measures that we want to, you know, train in, development, and some equality impact assessment work, heat tool to be used in our SOPs, just to bring everybody up to a baseline. So again, you can kind of measure that.” (CCDC-I-18022022) |
