# Supplementary material for: The role of health protection teams in reducing health inequities: findings from a qualitative study
Source: BMC Public Health. 2023 Feb 2;23:231. doi: 10.1186/s12889-023-15143-7 (PMC9893953; doi:10.1186/s12889-023-15143-7)
Supplement: Supplementary file 3 — Supplementary Material 3 [file 12889_2023_15143_MOESM3_ESM.docx]

Supplementary 3. Case studies of health protection health equity activities

“So it's around recognizing the difference between inequality inequity. So, for example, I can't remember exactly the details of it now, but essentially it was an infectious disease and there was someone who was homeless who had self-discharged… They had a mobile phone number associated with their thing. But, of course, they couldn't get through that mobile number and they weren't registered with a GP. I was having a conversation with one of my other consultant colleagues. They’re like, “well, we've done everything we usually do”. Now, my argument to that is, yeah, we have done everything that we'd usually do, but we're talking about someone who's particularly vulnerable here. So actually, this is where we I would expect us to go a little bit further. So, this isn't kind of a usual process. So, I guess, I feel that my responsibility is, particularly in kind of like a lead in health inequality, is to point out those times where equality and equity are actually two different things and we need to be focusing a little bit more on equity and kind of going that extra mile. … I think we have a real opportunity, and perhaps we don't do this, and that's not necessarily within our current kind of role and responsibility to kick off, kind of like trail process. So, for instance, you know we're informed that someone's in the hospital and they are a sex work or an intravenous drug user or homeless, and we’re informed that they've got invasive Group A strep. At that point, we know that they are at considerably higher risk of self-discharging, and they're almost certainly going to self-discharge before a point where their treatment is finished. Now, I think our role in that is, we’re being informed before lots of other people, because it's a notifiable disease. So, at that point, I believe it is our role to be cascading that information to the groups, that may be within their local authority, that may already be supporting these kind of individuals, to see if we can kind of link them up to try and keep them in hospital for as long as they need to have their treatment, or to ensure that they can receive their treatment in a way that's going to work for them. So, I think that that's kind of our role and responsibility.” (CCDC-I-17122021B)

“We'd got a case of hepatitis A in a child who was of primary school age and that was in a part of the **[Region] where there's 90 something percent of the school didn't speak English as their first language, which is actually quite uncommon up here in the **[Region]. So, rather than trying to communicate with the parents who travelled to somewhere abroad and that the child had got hepatitis A, we basically went down there the following day. The head teacher was amazing. They gathered all the parents together. They've got access to interpreters because of the population needs of that school and we did a presentation, and then the following day, with the school nursing team, we managed to get, I think, all of the children vaccinated against hepatitis A. So, we came together as a multi-agency with that school. That worked really, really well and it was nice to go out there into the community and be a part of that. And there was obviously access to people who could help support and interpret what we were saying. I think had that been a child in a school where they don't have access to all those facilities, trying to gather all that together within a couple of days just wouldn't have worked. So I think a proactive approach is going to be building back those relationships or continuing those relationships with all our partner organizations, certainly the school nursing teams. Schools, you know they definitely know who we are now, don't know they'd had to engage with us an awful lot. And I'd like to hope reactively and proactively, recovery on those strong bonds and they do come to us when they've got questions and when COVID has died down, so that they know to reach out to us .” (HPP-I-08032022)

“Pathways for patients with TB that were homeless. So, the first area focused on was for those with no recourse to public funds… So, we held a summit with about 90 partners. It was shortly after the national strategy was launched. We did loads of show and tell, good practice, bad practice and through that vehicle we agreed that one of our main priorities were this TB and homelessness, particularly those with no recourse to public funds, that there was no pathway to accommodation for these cases, so we were often losing cases or treating people on the streets and that, you know, it was not, you know, it didn't follow NICE guidance. It wasn't good patient care. So, we set up a small working group, which was chaired by one of the local authority public health colleagues, who was particularly passionate about this area of work. We did a review of the demand, so looked back at ETS and looked at how many cases of homelessness we'd had over a period of time. How many of those were related to no recourse to public funds, then looked at what pathways were in place and there was nothing that was robust. So, we did this sort of almost needs assessments, which one of the specialist registrars supported with and then we had to do some work with the directors of public health. So, we kind of took it back to the director of Public health; began a bit of a show and tell. And then with the proposal, we approached the **[Region] Accountable Officers Forum to look for funding to support these cases specifically based on the business case that you either stay in hospital for six months, which costs around £40,000 for an inpatient stay for that sort of duration. We had evidence of some cases that were in hospital for nine months because they had no accommodation, but were medically fit for discharge. And after a lot of sort of negotiation and relationship building and networking etc. we had four of the high incidence CCGs agree to co-fund this. We call it a risk share arrangements, based on the London model. It was more challenging for us sort of north of London because our accountable officers don't commission together on anything other than ambulances. So, whereas for the London office of CCGs, they do have a… Yeah, I don't understand the detail of it, but it was more challenging because we don't have that function. So, if you are looking to replicate this in other areas, it would be one of the things you need to consider. So, eventually they agreed to co-fund it. We set up operational policy, and then for two years we didn't have any cases at all. So, after making this big fuss about it, and then from September, I'm going to say 2020, I think we've had nine cases or 10 cases that have used. You know, it has been brilliant, particularly during the pandemic where things were so so challenging anyway. But for us to have this fund that we can draw down on, and the fund covers accommodation costs, a small living allowance, which is the same as the living allowance you get as a destitute asylum Seeker and travel expenses. And then, as part of that process we also linked them obviously into third sector support services in the local areas. So, they'll get food banks, food parcels and other sort of, not benefits, but other support from that sort of infrastructure in each of the local areas. Each area has done it slightly differently. So that's been a real challenge for us because providing support to people with no recourse is outside of what local authorities do, because they're not allowed to do that. So, there's been some real relationship challenges and had to do a lot of training and education and building teams within those local authorities. All the responses are local authority led, so they're a little bit different and we've hosted a couple of events to share learning. You know, when we're with the development of the ICS , the merger of the CCGs, some lower incidence areas have now joined that CCG, who've also now had some cases with NRPF. So, we've had to do some training and support to get them in a position to be able to respond, but it's worked really well and you know, it does obviously have its challenges and, clearly, you've got to have an exit strategy from the property when you complete treatment; so, you know, what's your exit strategy when you've still got no recourse to public funds? Is it to go back to your country of origin or is it to go back onto the streets? Is it to work and then fund your own living? Which many do. But, you know, we have a lot of obviously TB and the age group that it affects mainly are of working age. So, that has its challenges, but we're still learning and we need to update all of the paperwork and I'm happy to share anything once we've got. But as I say, it was based on the London model.” (PGM-I-09032022)

“viral hepatitis is seeking out inequalities wherever it goes. We had a massive outbreak of hepatitis A in men who have sex with men, which is a group who are much more exposed to sexually transmitted infections. Hepatitis B, massive inequalities. So for instance, I've done a lot of work on the system around vaccinating infants of hep B positive mothers who are now so-called women living with hep B, but haven't quite made the transition. So, we know about the inequalities there. We've got some really good epi data for it. It is about 10 years old, but it we don't think it's changed. For the pregnant women with hep B infection, like 40% have basic or less than basic English; they're mainly from non-white ethnic groups and they are often from groups that find it very hard to advocate for themselves, so both practical and, I am told by members of the Chinese community, also cultural reasons. So, it's more endemic in various pacific-rim countries. And so, I'm just trying to move from a system where those women have to advocate for their babies to be vaccinated, to one where the system offers and delivers and follows up. It has been a big piece of work. I've been involved with other hep B inequalities that I'm really trying to find a way to move on, but it's terrifically difficult for children in care and people with learning disabilities. So, I'm really interested in people with learning disability. They die much, I think it's 20 years younger. I might have the wrong number. They have vastly shortened life expectancies and there are Green Book recommendations about vaccinations for people who live in residential facilities for people with learning disabilities. And they're not implemented. I've got a documented case that's onsite transmission from one of those settings. I've got so many things I need to write up, but because this isn't like my main job, it's hard to do. And when I talk to the national team, they say you've got to write it up, because we need the evidence in the published literature. So, I think that's one of the problems is that this stuff comes up, but there's no time. So, as I say, that recommendation about adults with learning disabilities in residential care isn't implemented. Also, I've tried to get staff in special schools vaccinated against hep B where they get bitten a lot, and I would love to get the kids that bite a lot vaccinated, but it's super hard and that's probably the real problem is about commissioning arrangements for hep B vaccine. The other group is these children in care, children and young people, mostly 16 to 18. They're living in residential care facilities; supposedly supported, but you know it's not great. And these are kids with huge problems. And if you look at the list of ways to get hepatitis B and you look at what behaviors are more common in this group: unprotected sex, nonconsensual sex, getting into fights that draw blood, having tattoo not from a formal provider, injecting drugs, self-harming involving drawing blood. Those are all more common in that group. And so, when I've tried to follow that up with the national team, they've really interested and there isn't a bit of the organization that falls between both. So, both of those groups fall between. They're not in the formal inclusion health groups and so on. So that's a problem.” (CCDC-I-02032022)

“Work around trying to diversify representations of clinical signs in the medical literature. So, I first came across this through a medical student at St. Georges. And he basically did three weeks of medical studenting and then went to the staff and said, you know, your pictures don't help me make a diagnosis in my own family. He's Ghanaian heritage, I think. And it's his work. He put together a handbook for use in St. Georges… And there's a group at the Royal London called Skin Deep that's interested in inclusive dermatology… My hepatitis SOP still says hepatitis makes your skin go yellow and we know that 45% of the **[Region] population that we serve have given their ethnic group as not being white, so that's no good. I will update ours, but I thought we needed a process… So, I've linked up with a group and the core group is all people of colour… We've made our process for trying to do this, because I was really clear that we have to engage everyone, not just go and do something to people's SOPs. So we've now put it in the diary for the end of March between ourselves, we're calling it an engagement event… And we're going to try and get everyone on board with the project…. some pictures of rashes on different skin tones, and the descriptions from the SOP, just to show it, just very quick… And then asking people to come along on the journey. So, I think will get loads of volunteers. We need one from each group of SOP producers. We have local SOPs. And then the process we've all decided on is that: first we collect, you know, first discovery. First we collect the bits of text we think we need to fix, then we're going to put them all in an excel sheet with people's suggestions for replacements. But I was really clear that we then need to find some replacement text that's acceptable to everyone… thought it would be worth taking that kind of process to some more national inequalities groups too.” (CCDC-I-02032022)

“Things that we've initiated locally. We've done some work on infections in people who inject drugs, because that was noted to be a problem… We've done some work on putting together a training resource for hostels and people who work with that group of people, and some resources looking at what's in the needle exchange packs, with a focus on trying to prevent those bacterial infections as well as the blood borne virus prevention work.” (CCDC-I-10032022)

“I had an opportunity to think a bit more strategically about how, having got all of the epidemiological data to show who in **[HPT] was predominantly affected with TB, that we could then focus on working with particular groups. So, I had worked with a project manager and we facilitated focus groups with local community groups, particularly Chinese and Vietnamese communities with the local black African groups, women's refugee, health groups, prisons. I did a lot of work with prisoners and prison staff, actually, to sort of raise awareness around TB… We were linking up with a local community group around sort of trying to think of strategies to raise awareness and recognizing that this was a massively underserved group [talking about Somali population].” (CCDC-I-16022022)

“[FOCUS GROUP]

HPP-FG-24012022: And the recent one, actually from the Immigration hostel, there was an outbreak of chickenpox and you [speaking to someone else in the focus group] kindly translated the information of what is chicken pox in a different language… We've thought about translating leaflet sheets in other languages, especially if they don't speak English as their first language etc..

CCDC-FG-24012022: I think it's probably quite a nice example of the sort of different stages of where the health inequalities impact so and the particular population that we're talking about are recent arrivals, predominantly from Afghanistan, who don't have chicken pox immunity because it's very different epidemic in Afghanistan, or different levels of infection in Afghanistan toward it is in the UK, so that is normal for their population, but makes them more at risk when they come to the UK. Particularly, they've got children who are going in and out of schools; and, while a lot of them have been registered with a GP, their ability to access that GP is not simple, a) because there will be language barriers b) because they may just not understand how to. I think, you know, they're all registered very quickly to make sure that they were registered, but no one’s sat down and said this is what a GP is, this is when you see a GP, this is… And, you know, it's different in the UK to what it is in Afghanistan. How you access healthcare is different and this is how we do it here. And you need to know in case you need it. And also the ability of the GPs maybe to pick up all of the information required is not yet there, so while, if we had a chickenpox exposure, for example, in someone who's been living in this country for a very long time, we'd phone the GP and we say, do you have any evidence that this person ever had chickenpox? They say no, we don't, because it often doesn't go to the GP, we say, did you take booking bloods when they were pregnant? You know when they first got pregnant a couple of months ago? They say, yes we took booking bloods and we'd say, let's test those booking bloods, and we just go and test the booking bloods. But, of course, there aren't any booking bloods for the woman that's in initial accommodation. She hasn't had those assessments yet. It's all, so it's quite a good example of how… So, the part of the work that we impact is the kind of late stage. So, what do we do now? We translate all our information sheets. We facilitate a GP going in to assess whether, like, how many cases of chickenpox really are there. Are there people around her who are immunosuppressed? Do we need to be concerned about? Are there people who are pregnant we need to be concerned about? But we're working at that far end of the prevention, whereas actually, probably in the long term… is there something we can do a bit further beforehand, you know can we be working proactively, or with who is the organization we should be working proactively to provide that information about how you access a GP when you arrive?”

“This week I managed an outbreak of scabies in a hotel for asylum seekers, and I took that to the clinical meeting to draw out from that, the learning that scabies is not complicated and difficult, but 250 people, 13 different languages, no-one’s got a second outfit to change into once they had their treatment. That's the complicating factor and that's the inequalities issue. So, I took it to the clinical meeting, to do some learning and to draw out the idea that we look at people, not just disease; and particularly with Health Protection is that our advice goes into a context in which people are living. So, rather than saying, well, I just give out Health Protection advice, apply the cream and change your clothes. It's pointless to give out that advice unless you have considered the inequality issues: clothing and language and consent.” (CCDC-I-18022022)

“COVID and the vaccine uptake. So, we work with local communities within **[Region] to improve the uptake of COVID-19 vaccination. We have members who speak different languages, different cultural backgrounds, and we do video sessions with them. It's arranged by that health inequity group.” (HPP-I-02022022)

“There was a piece of work that I'd spoken to about someone in **[patch] saying that were things like Group A Strep, MRSA, PVL. It became clear that people up in **[city], that there were lots of people with drug use. There was a really high self-discharge rate, so they linked in with a local charity. They set up a wound clinic and they worked with the homeless team within the Council and they made like a one stop shop for people with drug and alcohol issues, so linked to that wound clinic that focused on drug and alcohol issues. Sexual health, housing, TB, etc. And the outreach team, kind of going out and helping get people off the street. So, there was like a pilot, was evaluated. It was done quite well, they said they did actually manage to carry on something through COVID.” (CCDC-I-17122021B)
